# Lactic Acid Bacteria Strains Exert Immunostimulatory Effect on *H. pylori*-Induced Dendritic Cells

**DOI:** 10.1155/2015/106743

**Published:** 2015-02-22

**Authors:** Małgorzata Wiese, Andrzej Eljaszewicz, Anna Helmin-Basa, Marek Andryszczyk, Ilona Motyl, Jolanta Wieczyńska, Lidia Gackowska, Izabela Kubiszewska, Milena Januszewska, Jacek Michałkiewicz

**Affiliations:** ^1^Collegium Medicum, Nicolaus Copernicus University, M. Sklodowskiej-Curie 9 Street, 85-094 Bydgoszcz, Poland; ^2^Department of Regenerative Medicine and Immune Regulation, Medical University of Białystok, Waszyngtona 13 Street, 15-269 Białystok, Poland; ^3^Faculty of Mechanical Engineering, University of Technology and Sciences in Bydgoszcz, Kaliskiego 7 Street, 85-789 Bydgoszcz, Poland; ^4^The Institute of Technology Fermentation and Microbiology, Faculty of Biotechnology and Food Sciences, Technical University of Lodz, Wólczańska 171/173 Street, 90-924 Łódź, Poland; ^5^Department of Clinical Microbiology and Immunology, Children's Memorial Hospital, Aleja Dzieci Polskich 20, 04-730 Warsaw, Poland

## Abstract

The aim of this study was to find out if selected lactic acid bacteria (LAB) strains (antagonistic or nonantagonistic against *H. pylori in vitro*) would differ in their abilities to modulate the DCs maturation profiles reflected by their phenotype and cytokine expression patterns. *Methods*. Monocyte-derived DCs maturation was elicited by their direct exposure to the LAB strains of *L. rhamnosus* 900 or *L. paracasei* 915 (antagonistic and nonantagonistic to *H. pylori*, resp.), in the presence or absence of *H. pylori* strain *cagA+*. The DCs maturation profile was assessed on the basis of surface markers expression and cytokines production. *Results*. We observed that the LAB strains and the mixtures of LAB with *H. pylori* are able to induce mature DCs. At the same time, the *L. paracasei* 915 leads to high IL-10/IL-12p70 cytokine ratio, in contrast to *L. rhamnosus* 900. *Conclusions*. This study showed that the analyzed lactobacilli strains are more potent stimulators of DC maturation than *H. pylori*. Interestingly from the two chosen LAB strains the antagonistic to *H. pylori-L. rhamnosus* strain 900 has more proinflammatory and probably antibactericidal properties.

## 1. Introduction 

Treatment of* H. pylori* infection is a long-term and not always efficient process. Antibiotic therapy leads to eradication of this pathogen in approximately 60–90% of the cases. However, even the efficiently treated individuals are still at risk of reinfection [1, 2]. Administration of selected strains of lactic acid bacteria (LAB), a component of intestinal microbiota, is an established factor improving efficiency of* H. pylori* eradication [3–5]. Some LAB strains prevent* H. pylori* colonization of gastric mucosa, thus decreasing the number of these bacteria in the stomach. The principle mechanism behind this effect of LAB is synthesis of lactic acid, which alters gastric pH and inactivates urease, a pivotal enzyme for* H. pylori* viability [6, 7]. The antagonism between LAB and* H. pylori* can be also associated with synthesis of other antibacterial compounds, for example, bacteriocins, autolysins, or thermostable proteins [7]. Apart from the bacterial antagonism, recent studies center around potential immunological mechanisms through which LAB can support eradication of* H. pylori* and attenuate inflammation of gastric mucosa. These include influence of LAB on enhanced local synthesis of IgA, modulation of specific IgG levels [8, 9], and induction of pro- and anti-inflammatory cytokine profiles [10].

Acute inflammation observed during an early phase of* H. pylori* infection is characterized by enhanced production of proinflammatory Th1/Th17 cytokines, presence of cell-mediated cytolysis, plasma cell infiltration, and synthesis of specific antibodies in the stomach and duodenum [11–14]. In turn, chronic inflammation associated with long-term gastrointestinal colonization by this pathogen is reflected by suboptimal Th1 response observed at later stages of the infection, as well as by an increase in Treg lymphocyte count [15–18]. The type of immune response is to a large extent determined by the activity of antigen-presenting cells (APCs), especially dendritic cells (DCs) which constitute a “link” between the nonspecific and specific responses [19–21]. Acute* H. pylori* infection is associated with migration of DCs to the antral mucosa [22–24]. The increased inflow of DCs during an early phase of inflammation results mainly from their ability to induce immune response against* H. pylori*. However, it is not reflected by elimination of this microorganism; phenotypic and functional changes of DCs result in development of chronic inflammation and tolerance of these cells to* H. pylori *antigens [15, 25, 26]. Therefore, two questions arose regarding whether this process could be modulated by intestinal microbiota, namely, by selected LAB strains, and whether antagonism between the latter bacteria and* H. pylori*, associated with release of antibacterial compounds, might modulate activity of the immune system. Moreover, still little is known on the immunological mechanisms associated with the development of* H. pylori *infection in presence of various strains of commensal bacteria [5, 7].

## 2. Material and Methods

### 2.1. Bacteria and Their Selection

The studied LAB strains were selected from among 29 strains of* Lactobacillus casei, Lactobacillus rhamnosus, Lactobacillus paracasei,* and* Lactobacillus plantarum*. The strains were identified by the sequencing of ribosomal RNA-encoding genes [27]. All the strains originated from the Pure Culture Collection of Industrial Microorganisms at the Technical University of Lodz (ŁOCK). The activity of interstrain antagonism was investigated using the agar slab method [28]. The method was based on the observation of parallel growth of the strains under study (the indicator—*H. pylori cagA+* strain 95 and one of the LAB strains). Agar slabs of 10 mm in diameter were aseptically cut off from the de Man, Rogosa and Sharpe medium (MRS, Oxoid) overgrown with a lawn of LAB strain incubated for 24 h at 37°C, 5% CO_2_, and placed on plates with Wilkins-Chalgren Anaerobe Agar (Oxoid) inoculated with the indicator strain (10^5^-10^6^ CFU/mL). After 5 days of incubation in anaerobic conditions at 37°C, the diameters of growth inhibition zones around the agar slabs were measured. The results are given in mm, minus the agar slab diameter (Table 1).

Finally, the study included human strains of two Gram-positive bacteria,* L. rhamnosus *900 and* L. paracasei* 915 (kindly provided by the Institute of Technology Fermentation and Microbiology, Faculty of Biotechnology and Food Sciences, Technical University of Lodz), and Gram-negative* H. pylori cagA+* strain 95 (obtained from the Department of Microbiology and Clinical Immunology, The Children's Memorial Health Institute). The isolated live bacterial strains or their combinations were used as stimulating agents in all the experiments.

### 2.2. Generation of Human Monocyte-Derived Dendritic Cells

Peripheral blood mononuclear cells (PBMCs) were isolated from buffy coat of healthy volunteers (from the Blood Centre in Bydgoszcz, Poland) by means of Lymphocyte Separation Medium 1077 (LSM, PAA) gradient centrifugation. Monocyte-derived DCs were generated from monocytes (CD14^+^ cells) isolated with an aid of CD14 beads (Becton Dickinson, positive selection), as previously described [29–32]. The purity of the cells was greater than 95%. Subsequently, the isolated cells (1 × 10^6^/mL) were cultured in RPMI 1640 (PAA) with 2% human serum (AB, Rh+ serum from the Blood Centre in Bydgoszcz, Poland) at 37°C and 5% CO_2_ for 6 days. IL-4 (50 ng/mL, R&D) and granulocyte-macrophage colony-stimulating factor (GM-CSF, 100 ng/mL, R&D) were added to the culture medium in order to stimulate DCs development.

### 2.3. Dendritic Cells Stimulation

The DCs (1 × 10^6^/mL) were suspended in 1 mL of RPMI 1640 (PAA) supplemented with 2% human serum (AB, Rh+ serum provided by the Blood Centre in Bydgoszcz, Poland) and incubated at 37°C and 5% CO_2_ in presence of* H. pylori, L. rhamnosus* 900,* L. paracasei* 915,* L. rhamnosus* 900 +* H. pylori,* or* L. paracasei* 915 +* H. pylori*. The DCs were incubated with bacteria or medium alone (control DCs) for 24 h. The DC to bacterial cell ratio was 1 : 10. The live bacteria at concentrations providing optimal maturity and viability of DCs (not shown) were used as stimulating agents in all the experiments. The cells were collected by gentle pipetting and centrifuged at 250 ×g for 10 min. The culture supernatant was collected and stored at −80°C until cytokine analysis. The cells were resuspended in PBS, and trypan blue exclusion test showed that the culture contained 90% of viable cells.

### 2.4. Cell Surface Phenotype Expression

Subsequently, the cells were stained for CD14, CD11c, CD80, CD86, and CD40 (all from Becton Dickinson) using mouse anti-human monoclonal antibodies conjugated with fluorescein isothiocyanate (FITC), phycoerythrin (PE), or peridinin-chlorophyll proteins (PercP). A total of 20 000 events were collected according to the manufacturer's procedure that was described elsewhere [33]. The cells were subjected to flow cytometric analysis with FACScan flow cytometer (Becton Dickinson), and the cytometric data were analyzed using FlowJo version 7.6.1 software (Tree Star). The percentage of cells showing expression of the studied receptors and the average receptor density expressed as the geometric mean of fluorescence intensity (GFI) were analyzed in a population of DCs.

### 2.5. Cytokine Assay

Cytokine concentrations in DCs cell culture supernatants were estimated following 24 h of bacterial or medium alone (control DCs) stimulation. The cytokine levels were measured by means of commercially available ELISA kits: DuoSet, BD Bioscience (IL-12p70, IL-10, and TNF-*α*), and R&D Systems (IL-23), according to the manufacturer's instructions. Before performing the tests, the supernatant samples were diluted according to each kit's protocol and the final results were obtained by appropriate multiplication. The protein level in the diluted sample was calculated from a reference curve generated for a given assay by using reference standards containing known concentrations of appropriate protein. Results were expressed as pg per mL. The range of cytokine detection was as follows: from 7.8 to 500 pg/mL for IL-12p70, IL-10, TNF-alfa and from 125 pg/mL to 8000 pg/mL for IL-23.

### 2.6. Statistics

Statistical analysis was conducted with Statistica 9.0 software (StatSoft). The normal distribution was checked using the Shapiro-Wilk test. Due to the nonnormal distribution of the data, Mann-Whitney *U* test was performed. Statistical significance was considered at *P* < 0.05.

## 3. Results

### 3.1. The Antagonistic Spectrum of LAB Strains

Antagonistic effect of LAB strains was tested against* H. pylori cagA+* strain 95. The antagonistic activity of* Lactobacillus* spp. was examined with the agar slab method, which is based on analysis of simultaneous growth of the indicator strain (*H. pylori cagA+* strain 95) and a tested strain (LAB). The results of the slab culture constituted the basis for selection of the studied strains of LAB. The strongest antagonistic effect against* H. pylori*, manifested by a 5.21 mm zone of inhibition, was documented in the case of* L. rhamnosus* 900. Finally, two strains of LAB were selected for further analyses:* L. rhamnosus *900, antagonistic to* H. pylori, *and the nonantagonistic* L. paracasei *915.

### 3.2. Phenotype of Monocyte-Derived DCs

**Figure 1:**
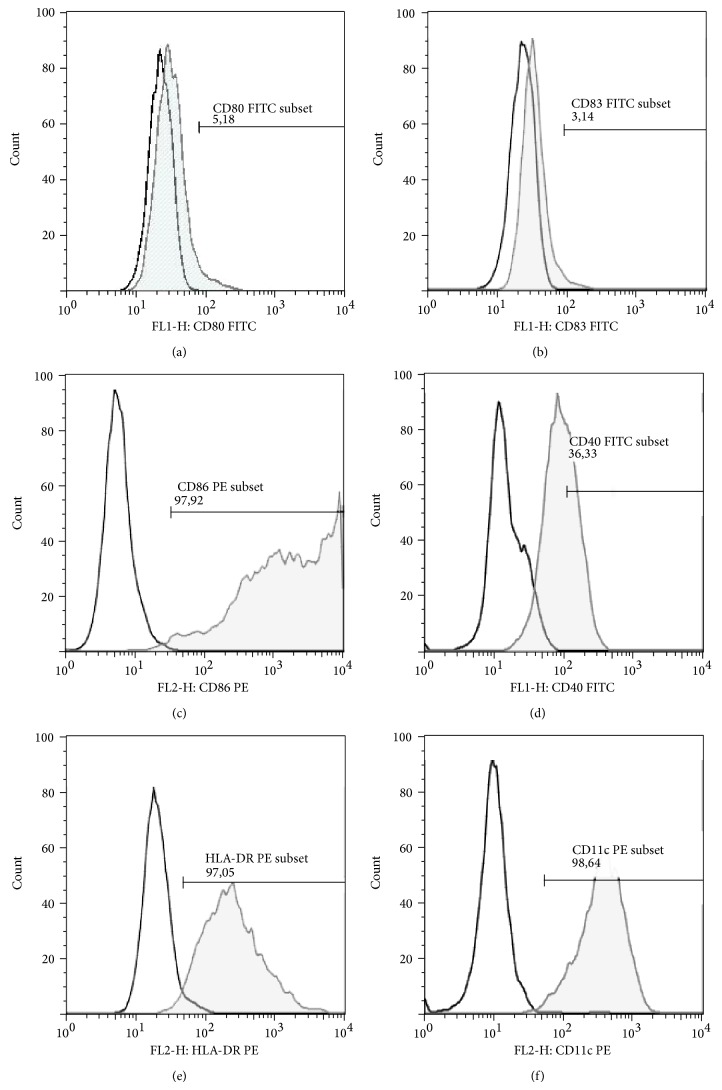
Phenotype of immature DCs. Histograms of representative cytometric data illustrate the following: (a) percentage of CD80^+^ DCs; (b) percentage of CD83^+^ DCs, (c) percentage of CD86^+^ DCs; (d) percentage of CD40^+^ DCs; (e) percentage of HLA-DR^+^ DCs; and (f) percentage of CD11c^+^ DCs. DCs: dendritic cells; stimulated DCs are represented by filled curves; isotype controls are represented by empty curves.

Monocyte-derived DCs were analyzed for surface phenotype by flow cytometry. Cells grown in GM-CSF and IL-4 alone after 6 days were immature, as defined by lack expression of CD14, relatively to stimulated DCs poor expression of CD83 and CD80 (Table 3) and lower expression of CD40, HLA-DR, and CD86. Almost all monocyte-derived DCs had expression of CD11c, characteristic marker for myeloid DCs (Figure 1, Table 2).

### 3.3. Phenotype of Bacteria-Stimulated DCs

Differences in the expression of DCs surface molecules were analyzed after one day of the bacterial stimulation (LAB strains:* L. rhamnosus 900*,* L. paracasei* 915;* H. pylori*; mixture:* L. rhamnosus *900 +* H. pylori* and* L. paracasei *915 +* H. pylori*) (Table 3).

Compared to the unstimulated DCs (control DCs), bacteria-stimulated DCs (irrespective of their variant) were reflected by a significant increase in HLA-DR and CD86 receptor densities on DCs (GFI for CD86 and GFI for HLA-DR).

Furthermore, the stimulation with either single bacterial strain caused a significant increase in the percentage of CD83-positive cells but the highest percentage of these cells was observed after stimulation with* L. paracasei* 915. A mixture of* L. paracasei* 915 +* H. pylori* turned out to exert stronger stimulatory effect on the expression of CD83-positive DCs than* H. pylori* alone or the mixture of* L. rhamnosus 900* and* H. pylori*.

A significant increase in the percentage of CD80-positive DCs was observed solely after exposure of DCs to LAB strains alone or in combination with* H. pylori.* In turn,* H. pylori* alone turned out to be significantly weaker inducer of the CD80-positive cells than the LAB strains and their mixtures. Moreover, we showed that exposure to* L. paracasei* 915 was reflected by significantly higher increase in density of CD80 receptor (GFI for CD80 receptor) than in the case of stimulation with* H. pylori*. Both* L. paracasei* 915 alone and in the mixture with* H. pylori* caused significantly greater increase in GFI for CD80 than did* L. rhamnosus* 900.

### 3.4. Comparison of Cytokine Levels after Bacterial Stimulation

**Figure 2:**
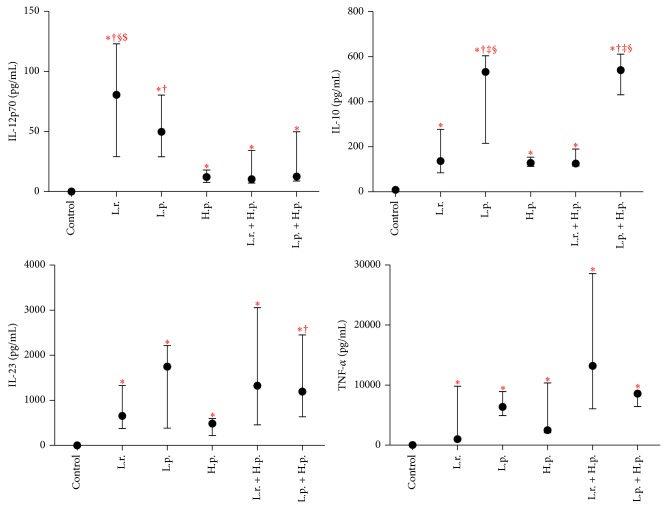
Effect of the examined bacteria and their mixtures on the production of cytokines by DCs population. Values expressed as medians from six independent experiments and interquartile ranges [Q1–Q3]; control: unstipulated DCs; L.r.:* L. rhamnosus *900; L.p.:* L. paracasei* 915; H.p.:* H. pylori*; statistically significant differences are given as follows: ∗: stimulators* versus* control (unstimulated DCs), †: stimulators* versus H. pylori, *‡: stimulators* versus L. rhamnosus *900, §: stimulators* versus L. rhamnosus *900* + H. pylori, *and $: stimulators* versus L. paracasei *915* + H. pylori*; *P* < 0.05; DCs: dendritic cells.

The DCs were stimulated for 24 h with live bacteria, either a single strain or a mixture of two bacterial strains (Figure 2). All the stimulators effectively induced cytokine synthesis (IL-10, IL-12p70, IL-23, and TNF-*α*) when compared with control DCs (unstimulated DCs).

*L. rhamnosus *900 alone turned out to be stronger inducer of IL-12p70 than* H. pylori* alone and mixtures of* H. pylori* + LAB. Also another analyzed LAB strain,* L. paracasei* 915, proved to be better stimulator of IL-12p70 synthesis than* H. pylori*.

Furthermore, the stimulation with either* L. paracasei* 915 alone or its combination with* H. pylori* was reflected by significantly more enhanced synthesis of IL-10 than the exposure to* L. rhamnosus* 900,* L. rhamnosus* 900 +* H. pylori,* and* H. pylori* alone.

Finally, stimulation of DCs with any of bacterial strains or their mixtures caused an increase in the synthesis of IL-23. However,* H. pylori* alone turned out to be a weaker stimulator of IL-23* versus L. paracasei* 915 +* H. pylori *(*P* < 0.05) and* L. rhamnosus *900 +* H. pylori* (*P* < 0.1).

Stimulation with all the bacteria and their mixtures resulted in a significant increase in TNF-*α* concentration, but without statistical differences.

**Figure 3:**
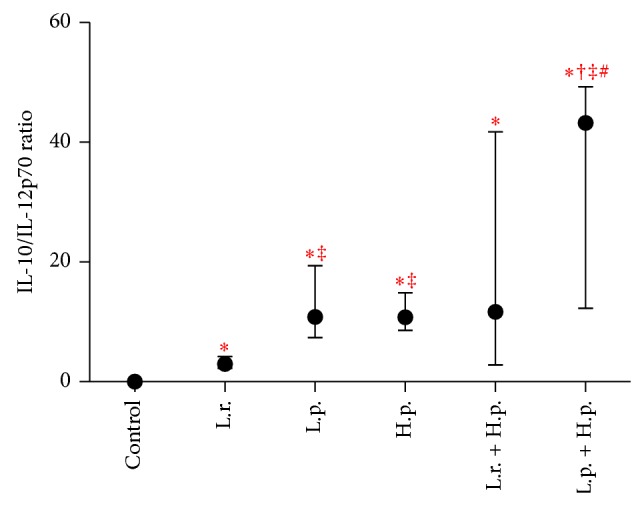
The cytokine IL-10/IL-12p70 ratio. Values expressed as medians from the ratios from six independent experiments and interquartile ranges [Q1–Q3]; control: unstipulated DCs; L.r.:* L. rhamnosus* 900; L.p.:* L. paracasei* 915; and H.p.:* H. pylori*; statistically significant differences are given as follows: ∗: stimulators* versus* control (unstimulated DCs), †: stimulators* versus H. pylori, *‡: stimulators* versus L. rhamnosus *900, stimulators* versus L. paracasei* 915; *P* < 0.05; DCs: dendritic cells.

Next, we calculated the IL-10/IL-12p70 ratios obtained from these studies (Figure 3). These allowed the ranking of the strains from an “anti-inflammatory” to a “proinflammatory” profile. The strains* L. paracasei* 915 and* H. pylori* were classified as more anti-inflammatory.* L. rhamnosus* 900 showed a slightly proinflammatory profile with a very low IL-10/IL-12p70 ratio. The mixture* L. paracasei* 915 +* H. pylori* showed strong anti-inflammatory capability. In contrast, despite the rather high ratio of IL-10/IL-12p70, the mixture* L. rhamnosus* 900 +* H. pylori* did not show differences between stimulators.

## 4. Discussion

In this study, we provided evidence for the immunostimulatory effect of LAB strains on* H. pylori*-induced DCs. We also reported for the first time that the LAB strains induce more mature phenotype of DCs than* H. pylori* alone (as shown by greater percentage of CD80^+^ DCs). Thus, our findings point to potential application of some of these bacteria as a component of* H. pylori* infection treatment.

There are three consecutive stages of DC maturation: immature DCs (iDCs), semimature DCs (smDCs), and mature DCs (mDCs). The cells representing these phenotypes can be distinguished on the basis of cytometric analysis of HLA-DR, CD80, CD86, CD83, and CD40 receptor expressions and profile of secreted cytokines, such as IL-10, IL-12p70, IL-23, and TNF-*α* [34–36]. Activation of iDCs with foreign antigens, for example, bacterial Ag, results in their transformation to smDCs or mDCs. The phenotype of semimature DCs does not differ from that of mDCs: their ability to synthesize cytokines is limited as shown by markedly lower concentrations of proinflammatory cytokines and moderate level of IL-10 in culture supernatant. In contrast, the fully mature DCs cause activation of T cell response and synthesize an array of cytokines, for example, IL-12p70, IL-12p40, IL-6, and TNF-*α* [34, 35, 37]. It is noteworthy that all DCs constitutively express CD86 and HLA-DR on their surfaces [38, 39]. Therefore, we identified iDCs, mDCs, and smDCs on the basis of percentage of cells expressing CD83 and CD80 and fluorescence intensity of these receptors on DC surface as well as cytokine production.

The increase in the percentage of CD83^+^ cells, observed after stimulation with either all the analyzed bacteria (*H. pylori*, LAB) or their mixtures (LAB with* H. pylori*), likely reflected the process of DC maturation [31, 40].* L. paracasei *915, that is, the strain nonantagonistic to* H. pylori*, turned out to be the most potent activator of DC maturation among all the analyzed variants, as shown by the most pronounced increase in the percentage of CD83^+^ cells and density of CD80. Analysis of the expression of CD80 receptor, responsible for late activation of DCs [38], showed that* H. pylori* was the only bacterium that did not stimulate an increase in the percentage of CD80^+^ cells. Therefore, the analyzed strain of* H. pylori* stimulated maturation of DCs to a lesser extent, which likely corresponded to development of smDCs with tolerogenic phenotype [41, 42]. In contrast, the mixtures of* H. pylori* with the LAB strains stimulated differentiation of CD80-positive DCs. Therefore, the analyzed lactobacilli likely enhanced the process of DCs maturation despite the presence of* H. pylori*. This phenomenon may directly affect the following: (a) presentation of Ag to antigen-naive lymphocytes T, (b) profile of secreted cytokines, and (c) characteristics of T-dependent response (e.g., predominance of Th1, Th2, or Th17 response).

It is commonly known that the effective response of T lymphocytes requires two types of activation signal: (a) interaction between Ag presented by MHC I/II and the TCR/CD3 receptor and (b) interaction between receptors, such as CD80, CD86, and CD28 or CTLA-4. Too weak second signal leads to anergy of T lymphocytes and resultant apoptosis thereof [38, 43]. The abovementioned process involves a number of molecules supporting the presentation, such as CD40 and CD83 participating in activation of T lymphocytes [44, 45]. Although we documented an increase in the percentage of CD83^+^ cells in DC population, both relative and absolute numbers of CD40^+^ cells remained unchanged. Moreover, it should be stressed that all the analyzed strains and the mixtures thereof exerted similar effect on CD86 and HLA-DR expressions. These findings suggest that we did not obtain fully mature DCs since, as mentioned previously, the presence of the latter needs to be confirmed by secretion of specific cytokines to culture supernatant. Apart from maturation of DCs, also polarization of these cells toward DC1 or DC2 function constitutes equally important component of response to* H. pylori* infection; the process of polarization can be analyzed on the basis of concentrations of selected cytokines, especially IL-12p70 and IL-10. The fact that the level of biologically active form of IL-12 after stimulation with* H. pylori* alone was lower than after the exposure to the analyzed LAB strains may reflect immunosuppressive effect of* H. pylori* or polarization of DC towards Th2 response [15]. It should be emphasized that the mixtures of analyzed LAB strains (*L. rhamnosus* 900 and* L. paracasei *915) and* H. pylori *induced secretion of IL-12p70 at a similar level as did* H. pylori* alone, which suggests that the latter bacterium might inhibit the LAB-induced immune response. This hypothesis is supported by the results of a previous study in which* H. pylori* was shown to release a factor that inhibited secretion of IL-12 by DCs [24, 46]. However, despite the fact that IL-23 belongs to the family of IL-12, similar effects were not observed. We showed that the level of IL-23 after stimulation with* L. paracasei* 915 and* H. pylori* mixture was significantly higher than in the case of exposure to* H. pylori* alone. In turn, the concentration of IL-23 in the culture of DCs stimulated with the mixture of* L. rhamnosus* 900 and* H. pylori *turned out to be similar as in the case of DCs induced with* L. paracasei* 915 and* H. pylori*. High level of IL-23 corresponds to proinflammatory function of activated DCs and can be associated with induction of Th17 response [47, 48]. The DCs stimulated with the bacterial mixtures seemed to be more effective and their phenotype resembled that of mDCs to a larger extent than the phenotype of the cells exposed to* H. pylori *alone. Enhanced synthesis of IL-23, involved in the control of Th17 response, may be beneficial in the case of* H. pylori*-induced inflammation as previous studies showed that it improves the antibacterial potential [13, 22]. Apart from IL-23, DCs synthesize an array of other proinflammatory cytokines, for example, TNF-*α* [49]. Both LAB and* H. pylori*, as well as their mixtures, enhanced synthesis of TNF-*α*; however, the levels of this cytokine did not differ significantly between the analyzed culture variants. Previous studies showed that bacterial stimulation of DCs is reflected by enhanced synthesis of TNF-*α*; this cytokine exerts pleiotropic effects [50–55], determined by duration of the exposure. Moreover, high level of TNF-*α* was shown to be a marker of DC maturation. It is interesting that mature DCs can also synthesize these cytokines that act antagonistically to proinflammatory cytokines, for example, IL-10 [56]. Both* L. paracasei* 915 strain and the mixture thereof with* H. pylori* turned out to be the strongest inductors of IL-10 synthesis. These findings confirm that a nonantagonistic strain can stimulate tolerogenic response associated with activation of type-2 polarized DCs. The concentration of IL-10 documented after stimulation with* H. pylori* was markedly lower, similar to that observed after exposure to* L. rhamnosus *900 alone or in mixture with* H. pylori.* As mentioned above, low level of this cytokine may be characteristic for smDCs [34], which further confirms that stimulation with* H. pylori* promotes tolerogenic phenotype of DCs. Low levels of both IL-10 and IL-12p70 in* H. pylori*-induced culture may also point to the lack of DC polarization and result in the lack of their reactivity with T lymphocytes. However, the hereby presented findings suggest that such dysregulation of immune response may be at least partially counterbalanced by LAB strains, as shown by increased expression of DC surface markers (CD80 and/or CD83) and higher concentration of IL-23 in culture supernatant.

The fact that LAB stimulated maturation of DCs suggests that these bacteria may normalize immune mucosal function during symptomatic* H. pylori* infection. However, we could not unambiguously distinguish which of the LAB strains, antagonistic or nonantagonistic one, was a stronger enhancer of antibacterial reaction associated with activation of T-dependent (Th1, Th17) response. On one hand, we documented a marked increase in CD80 expression solely on the surface of DCs stimulated with* L. paracasei* 915 and its mixture with* H. pylori*, which points to greater potential of the nonantagonistic LAB strainas a stimulator of DC maturation. On the other hand, the same LAB strain proved to be a strong inductor of IL-10 synthesis. In turn, this cytokine is known to stimulate response of Treg lymphocytes [57], and percentage of these latter cells increases in the course of* H. pylori* infection, being tightly associated with the activity and phenotype of DCs. In contrast, elimination of Tregs may promote eradication of* H. pylori* [58]. Therefore, lower mucosal counts of Tregs will be reflected by stronger immune response (Th1 or Th17 response) and resultant elimination of* H. pylori*. Understanding the profile of T lymphocyte in the coculture of these cells with LAB/*H. pylori*-stimulated DCs is warranted (actually under study). However,* L. rhamnosus* 900 in contrast to* L. paracasei* 915 shows reduced IL-10/IL-12p70 ratio. Therefore, it seems that nonantagonistic strain may be more supressive/tolerogenic. It should be noted also that the mixture* L. paracasei* 915 +* H. pylori *was also strongly anti-inflammatory. Taking together,* L. rhamnosus* 900 proved to be a weaker stimulator of DC maturation, the polarization of cellular response induced by this bacterium could be more beneficial in the context of* H. pylori* infection.

## 5. Conclusions

First, the LAB strains used here were much more potent DC maturation agents than* H. pylori*. Second,* H. pylori*-induced DCs tolerogenic phenotype was at least partially overcome by the LAB strains. Third, the* L. rhamnosus* strain 900 (antagonistic to* H. pylori*) proved to be more effective than* L. paracasei* strain 915 (nonantagonistic to* H. pylori*) in DCs protection against tolerogenic action of* H. pylori*.

## Figures and Tables

**Table 1:** Antagonistic activity of lactic acid bacteria strains.

Species	Strain	Growth inhibition zone [mm]
*Lactobacillus casei *	ŁOCK 899	1.7
	ŁOCK 901	0
	ŁOCK 902	0
	ŁOCK 903	0
	ŁOCK 904	0
	ŁOCK 905	1.7
	ŁOCK 906	2.1
	ŁOCK 907	0
	ŁOCK 908	4.8
	ŁOCK 909	0
	ŁOCK 910	0
	ŁOCK 911	2.5

*Lactobacillus rhamnosus *	ŁOCK 900^*^	5.21

*Lactobacillus paracasei *	ŁOCK 912	0
	ŁOCK 913	2.9
	ŁOCK 914	0
	ŁOCK 915^*^	0
	ŁOCK 916	2.9
	ŁOCK 917	1.6
	ŁOCK 918	2.7
	ŁOCK 919	3.6
	ŁOCK 920	2.2
	ŁOCK 921	1.7
	ŁOCK 922	0
	ŁOCK 923	0
	ŁOCK 924	0

*Lactobacillus plantarum *	ŁOCK 862	2.3
	ŁOCK 864	0
	ŁOCK 943	1.6

^*^The selected strains.

**Table 2:** Expression of chosen receptors (CD14, HLA-DR, CD80, CD83, CD86, CD40, and CD11c) on monocyte-derived DCs surface.

Receptor type	CD14	HLA-DR	CD80	CD83	CD86	CD40	CD11c
iDC							
%	1.98 [0.38–1.99]	97.11 [89.30–99.55]	4.99 [2.88–5.73]	1.27 [0.89–3.32]	98.55 [97.25–99.70]	26.4 [12.10–37.95]	98.7 [97.50–99.00]
GFI	183 [146–220]	336 [274–363]	181 [170–188]	150 [145–160]	463 [350–581]	160 [130–190]	676 [650–793]

DCs: dendritic cells; iDCs: immature DCs; %: the percentage of DCs expressing the analyzed receptor; GFI: geometric mean fluorescence intensity of the analyzed receptor in DCs population exhibiting its expression; values are expressed as the medians of six independent experiments and range of lower quartile-upper quartile [Q1–Q3].

**Table 3:** Effect of the examined bacteria and their mixtures on the expression of DCs markers (CD14, HLA-DR, CD80, CD83, CD86, and CD40).

Receptor type	Control	*L. rhamnosus *900	*L. paracasei *915	*H. pylori *	*L. rhamnosus *900 + *H. pylori *	*L. paracasei *915 + *H. pylori *
CD14						
%	0.83 [0.47–0.87]	1.57^ ^[0.38–2.83]	0.89 [0.50–2.67]	1.29 [0.85–2.18]	1.63 [0.43–1.92]	0.85 [0.41–4.70]
GFI	172 [147–221]	217 [165–230]	198 [162–256]	162 [149–179]	206 [157–211]	232 [169–258]
HLA-DR						
%	95.42 [94.80–99.21]	96.21 [94.90–98.11]	98.21 [95.60–98.50]	98.70 [98.60–99.15]	96.85 [95.09–97.30]	96.75 [94.30–99.11]
GFI	335 [294–360]	778^*^ [700–1806]	765^*^ [632–1423]	1504^*^ [1040–1811]	777^*^ [717–1989]	839^*^ [713–1436]
CD80						
%	4.20 [3.91–5.18]	15.50^∗†^ [12.40–28.20]	37.80^∗†^ [25.80–47.60]	3.71 [2.53–11.90]	18.5^∗†^ [13.10–29.10]	36.65^∗†‡^ [28.10–56.20]
GFI	154 [140–181]	152 [147–160]	216^∗†‡^ [166–224]	154 [149–162]	149 [148–170]	177^‡^ [161–185]
CD83						
%	1.56 [0.99–1.94]	14.7^*^ [8.95–18.88]	39.35^∗†‡§^ [27.27–45.00]	6.27^*^ [3.94–13.01]	13.35^*^ [9.52–18.43]	30.65^∗†§^ [18.77–50.98]
GFI	160 [146–207]	155 [132–239]	152 [141–157]	147 [140–159]	155 [155–181]	143 [136–152]
CD86						
%	97.25 [89.11–99.61]	96.45^ ^[95.18–99.10]	96.30 [95.66–99.43]	99.00 [95.44–99.33]	97.45 [95.71–99.55]	97.12 [94.96–99.35]
GFI	554 [373–608]	2740^*^ [1118–3390]	2079.5^*^ [899–3143]	3388^*^ [1514–3742]	3447^*^ [3048–3667]	2496^*^ [2002–3226]
CD40						
%	35.51 [17.33–40.41]	30.26^ ^[26.34–39.89]	23.70 [23.46–27.78]	21.05 [16.95–28.25]	30.45 [24.79–53.67]	26.65 [12.17–45.45]
GFI	145 [105.2–175]	145 [141–153]	175 [132–219]	131 [123–140]	146 [127–167]	145 [142–149]

DCs: dendritic cells; %: the percentage of DCs expressing the analyzed receptor; GFI: geometric mean fluorescence intensity of the analyzed receptor in DCs population exhibiting its expression; values are expressed as the medians of six independent experiments and range of lower quartile-upper quartile [Q1–Q3]; statistically significant differences: ^*^stimulators *versus* control (unstimulated DCs), ^†^stimulators *versus  H. pylori*, ^‡^stimulators *versus L. rhamnosus 900*, and ^§^stimulators *versus L.rhamnosus *900* + H. pylori*; *P < 0.05.*
